# Supplementary material for: Conditional chemoconnectomics (cCCTomics) as a strategy for efficient and conditional targeting of chemical transmission
Source: eLife. 2024 Apr 30;12:RP91927. doi: 10.7554/eLife.91927 (PMC11060718; doi:10.7554/eLife.91927)
Supplement: Figure 3—source data 3. [file elife-91927-fig3-data3.zip › Figure 3 source data3 part 1.zip!/Figure 3-Source data 3-part1/1082-9-CRISPResso_Report_zod2ewbs.zip!/CRISPResso_on_1082_12/9.Alleles_frequency_table_around_sgRNA_catccaggcagatccaggtg.pdf]

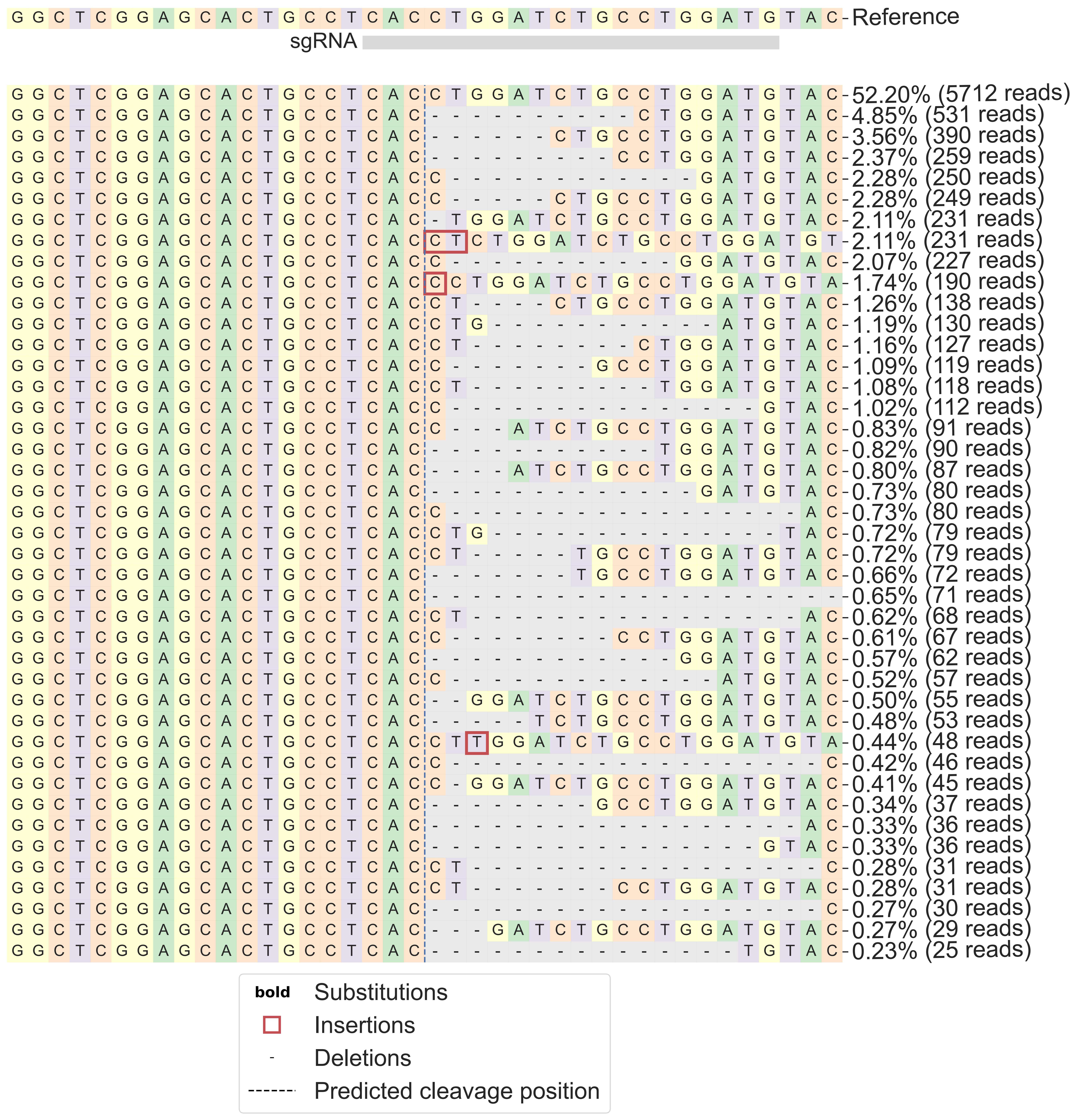
Reference
sgRNA
52.20% (5712 reads)
4.85% (531 reads)
3.56% (390 reads)
2.37% (259 reads)
2.28% (250 reads)
2.28% (249 reads)
2.11% (231 reads)
2.11% (231 reads)
2.07% (227 reads)
1.74% (190 reads)
1.26% (138 reads)
1.19% (130 reads)
1.16% (127 reads)
1.09% (119 reads)
1.08% (118 reads)
1.02% (112 reads)
0.83% (91 reads)
0.82% (90 reads)
0.80% (87 reads)
0.73% (80 reads)
0.73% (80 reads)
0.72% (79 reads)
0.72% (79 reads)
0.66% (72 reads)
0.65% (71 reads)
0.62% (68 reads)
0.61% (67 reads)
0.57% (62 reads)
0.52% (57 reads)
0.50% (55 reads)
0.48% (53 reads)
0.44% (48 reads)
0.42% (46 reads)
0.41% (45 reads)
0.34% (37 reads)
0.33% (36 reads)
0.33% (36 reads)
0.28% (31 reads)
0.28% (31 reads)
0.27% (30 reads)
0.27% (29 reads)
0.23% (25 reads)
bold Substitutions
Insertions
Deletions
Predicted cleavage position
